# Dermatitis Herpetiformis: From the Genetics to the Development of Skin Lesions

**DOI:** 10.1155/2012/239691

**Published:** 2012-06-07

**Authors:** Diletta Bonciani, Alice Verdelli, Veronica Bonciolini, Antonietta D'Errico, Emiliano Antiga, Paolo Fabbri, Marzia Caproni

**Affiliations:** ^1^Section of Dermatology, Department of Medical and Surgical Clinical Care, University of Florence, 50129 Florence, Italy; ^2^Department of Clinical Physiopathology, University of Florence, 50139 Florence, Italy

## Abstract

Dermatitis herpetiformis (DH) is a rare autoimmune disease linked to gluten sensitivity with a chronic-relapsing course. It is currently considered to be the specific cutaneous manifestation of celiac disease (CD). Both conditions are mediated by the IgA class of autoantibodies, and the diagnosis of DH is dependent on the detection of granular deposits of IgA in the skin. There is an underlying genetic predisposition to the development of DH, but environmental factors are also important. This paper describes these different factors and discusses the known mechanism that lead to the development of skin lesions.

## 1. Introduction 

Dermatitis herpetiformis (DH) is an autoimmune disease linked to gluten sensitivity with a chronic-relapsing course, characterized by pruritic polymorphic lesions and typical histopathological, immunopathological, and serological findings. It is currently considered to be the specific cutaneous manifestation of celiac disease (CD) [1]. 

Patients with DH and CD share many common features such as gluten sensitivity, the same strong human leukocyte antigen (HLA) association, the presence of circulating IgA antitissue (tTG), and epidermal transglutaminase (eTG) antibodies [2]. Moreover, both DH and CD show the same typical histologic features of villous atrophy of the small bowel. In DH, the spectrum of enteropathy varies, and 20% of patients show apparently normal small-bowel mucosal architecture, but there are virtually always inflammatory changes consistent with latent CD [3, 4]. 

Both the rash and the enteropathy improve after a gluten-free diet (GFD) [5]. 

DH presents with diffuse, symmetrical, grouped polymorphic lesions consisting of erythema, urticarial plaques, papules, herpetiform vesiculae, and blisters followed by erosions, excoriations, and hyperpigmentation [6–9]. The most commonly involved sites are the elbows (90%), knees (30%), shoulders, buttocks, sacral region, and face. Itching of variable intensity, scratching, and burning sensation immediately preceding the development of lesions are common [6–9].

The presence of granular deposits of IgA at the tips of the papillary dermis is considered highly suggestive of the disease [10], even if DH may have a fibrillar rather than granular pattern of IgA deposition on direct immunofluorescence (DIF) microscopy, and patients with this pattern may lack circulating autoantibodies [11].

Although DH is a rare disease, it is more common in Caucasians, while it is rarer in Asian populations, including the Japanese. Several differences between Caucasian and Japanese DH are reported, such as a higher frequency of the fibrillar pattern, a rarer gluten-senstiive enteropathy (GSE), and different HLA haplotype in Japanese [12]. 

The pathophysiology of DH is complex and involves genetic factors, such as HLA predisposition, environment trigger (gluten), and disregulation of the immune system [13]. 

## 2. Genetic Factors 

As in CD, virtually all patients with DH carry either HLA DQ2 or HLA DQ8 haplotypes [14]. This association has been demonstrated both in human and animals models. 

In a study by Spurkland, comparing 50 patients with DH to 289 healthy controls, 86% of affected patients carried the HLA DQ2 allele and 12% carried the HLA DQ8 allele. The presence of either of both alleles provides a sensitivity of close to 100% for CD and DH. In individual lacking these alleles, CD and DH are virtually excluded [15]. 

NOD DQ8+ murine models reproduced by Marietta et al. have confirmed these associations. Fifteen NOD DQ8+ mice out of 90 that were sensitized to gluten developed blistering pathology similar to that seen in DH. Accordingly, neutrophil infiltration of the dermis, deposition of IgA at the dermal-epidermal junction (DEJ), and a complete reversal of the blistering phenomenon with the administration of a GFD with or without dapsone were observed [16]. Although it was a gliadin immunocomplex disease model rather than an experimental DH, such a model emphasized the role of HLA-DQ8 haplotype. In fact, according to the authors, the addition of DQ8 contributes sensitivity to gliadin, while the addition of the NOD background contributed to the autoimmune diathesis.

Previous genetic studies conducted in the 1970s and 1980s showed an increased expression of the HLA-A1 [17], HLA-B8 [18, 19], and HLA-DR3 [20] haplotypes in patients with DH and CD. For HLA-B8, the association with DH was 58–87% versus 20–30% for control patients [18, 19]. For HLA-DR3, the association with DH was 90–95% versus 23% for control patients [21]. However, these associations have not been subsequently confirmed by further studies, and they do not seem statistically significant in relation to the HLADQ2 and HLA DQ8 haplotypes.

Several studies showed differences in HLA haplotype between Caucasian and Asian patients. In particular in Japan, the HLA-B8/DR3/DQ2 frequency is very low in the general population (1% or less), and no HLA-B8/DR3/DQ2 haplotypes were found in DH patients [22].

Many studies emphasized that genetic factors, other than HLA, play an important role in the pathogenesis of DH [23–28]. A high concordance was demonstrated in monozygotic twins [23] (concordance ratio 0.91), and the incidence of both CD and DH is higher among first-degree relatives than that in general population [24]. 

Recently, a novel candidate gene, myosin IXB (*MYO9B*) on chromosome 19p13, was shown to be associated with CD in the Dutch and Spanish populations [25, 26]. Myosin IXB functions in cell signalling and regulation of actin cytoskeleton dynamics, thereby regulating cell integrity and permeability of the gut barrier. In patients with myosin IXB mutations, there could be an increased permeability of the intestine with more gluten penetration and a subsequent immunologic triggering results in clinically overt CD or DH [27].

Koskinen et al. [28] studied linkage and association of four *MYO9B* single-nucleotide polymorphisms (SNPs) on chromosome 19p13 with CD in a total of 1259 patients with CD; 161 (13%) of them suffered from DH. 

The results showed significant linkage to 19p13 which supports the presence of a genuine risk factor for CD in this locus, while weak evidence of association with DH was found. In fact, when the family material of patients with the disease was divided into two groups according to the occurrence of DH, the *MYO9B* risk SNP alleles were found to be significantly overtransmitted to the offspring in only the families with DH [28].

## 3. Environmental Factors 

Environmental trigger factors for DH are essentially represented by the ingestion of gluten (a glycosylated storage protein of cereals like wheat, rye, and barley), the same component whose addition or removal can turn the disease process on and off in CD [29, 30]. DH and CD are significant examples where an environmental factor plays a central role in the pathogenesis of the disease [31]. 

Gliadins are the alcohol soluble part of gluten with particularly high content of glutamine and proline [32]. Gliadins are only partially digested in the gut comprising peptides which are resistant to digestion [33]. Such digestion-resistant peptides can thus be modified by tTG in two alternative ways that include deamidation and transamidation [34, 35]. *In vitro* studies have demonstrated that transamidation is the major reaction thus increasing the antigenicity of gliadin peptides [36]. Neoantigens are created by these enzymatic modifications of dietary gluten. Cross-linking also outside the active site of tTG results in permanently and covalently linked deaminated gliadin/peptide/tTG complexes [37, 38] which are found in small intestine biopsies of patients with CD [39]. 

Gliadins are separated according to their electrophoretic mobility into four groups (*α*, *β*, *γ*, and *δ*), each one is further subdivided into other fractions [40]. It has been shown that the fraction most involved in bowel mucosa lesions is gliadin A, the main constituent of *α*-gliadins [41, 42]. The primary amino acid structure of this subfraction is known, and it is believed that its immunoreactive activity is related to the N-terminal region of the molecule [43]. 

The most convincing demonstration that gluten, as in CD, represents the etiological factor in DH is the finding that a strict GFD, even if after many months, can resolve cutaneous manifestations, in addition to the intestinal disorder [44, 45]. Several lines of evidences support the interpretation that the gastrointestinal alterations induced by *α*-gliadin are also able to trigger the mucosal immunoresponse, which is not limited to this antigen but is extended to tTG and moreover to eTG, with a title related to the entity of intestinal damage [46]. 

## 4. Antitransglutaminase IgA Antibodies 

Until the 90s, circulating IgA EMAs were considered the most important serologic marker both in DH and in CD [47]. In 1997, Dieterich et al. identified tTG as the unknown endomysial autoantigen in CD [48]. tTG is a primarily cytoplasmatic, calcium-dependent enzyme that catalyzed cross-linking between glutamine and lysine protein residues [49]. tTG is ubiquitously expressed in many tissues; in the skin, it is found in the basal keratinocytes and dermal capillaries [50]. 

The presence of circulating anti-tTG IgA is the most sensitive marker for CD, and it is commonly used as a screening tool [51]. Anti-tTG IgA antibodies are also diagnostic markers for enteropathy in DH patients [52]; their levels, in fact, correlate with the degree of intestinal damage and decrease under a GFD [53]. Therefore, levels of tTG-specific antibodies serve as a useful indicator of patient adherence to a GFD [54, 55]. 

In 2002, another autoantigen, namely eTG, was identified for DH [56]. eTG is homologous of tTG within enzymatically active domains [50, 57], and its main function in the epidermis involves cross-linking and the maintenance of cornified envelope integrity [57, 58]. eTG is not ubiquitary expressed, but it is primarily seen in the epidermis, small intestine, brain, and testis [58]. 

In 2002, Sardy et al. [56] demonstrated that the IgA deposits in the perilesional skin of DH patients colocalize with eTG deposits. Recently, Donaldson et al. [59] and Marietta et al. [60] independently confirmed these results in two different studies.

Patients with DH produce two IgA antibody populations against eTG. The first population binds exclusively eTG, whereas the second one cross-reacts with both eTG and tTG [53]. The cross-reactive eTG-specific antibodies are found in CD without DH yet, but they demonstrate a lower avidity for eTG than in patients with DH. In contrast, eTG-specific antibodies non-cross-reactive with tTG are found only in patients with DH [56]. Furthermore, eTG but not tTG colocalizes with granular IgA deposits in the skin of patients with DH [56, 59], and levels of antibodies against eTG correlate with the extent of enteropathy in DH but not in CD without DH [60]. Taken together, these data suggest that eTG rather than tTG seems to be the autoantigenic target in patients with DH, while tTG is the dominant antigen for CD. A study by Rose et al. [53] demonstrated that antibodies to eTG are the most sensitive serologic marker for the diagnosis of DH. In particular, a sensitivity of 95% was reported, confirming the study by Sardy et al. [56], in which the sensitivity was 92%, although Heil et al. [61] and Hull et al. [62] found anti-eTG antibodies in only 45% and 52% of patients with untreated DH, respectively. 

Basing on these data, DH could be seen as a cutaneous IgA-eTG immunocomplex disease, developing only in a few patients with gluten-sensitive enteropathy. In agreement with this view, a histopathological pathogenetic model has been recently suggested by Zone and coworkers [63], trying to explain the onset of DH. They proposed that, during childhood, patients with CD initially have elevated levels of IgA anti-tTG antibodies but normal levels of IgA anti-eTG antibodies. As time goes on and patients with CD become adults, some of them, probably due to “intermoleculary epitope spreading,” develop elevated levels of IgA anti-eTG antibodies compared with their childhood counterparts. It has been hypothesized that these patients who develop elevated level of IgA anti-eTG antibodies in adulthood are at high risk of developing DH. 

However, though interesting, such a model should be confirmed by further studies. In fact, DH is not uncommon in children, but according to the theory by Zone, they would not have had time to undergo epitope spreading and develop anti-eTG antibodies [64]. 

Confirming the role of anti-eTG in the pathogenesis of DH, Zone et al. demonstrated that passive transfer of both goat IgG anti-eTG and human IgA anti eTG antibodies in SCID mice bearing human skin grafts induces granular IgA deposition in dermal papillae of human skin in a pattern similar to that of DH. However, mice did not develop inflammatory lesions with either IgG or IgA anti-eTG antibodies transfer, suggesting that additional proinflammatory events could be required for the eventual development of inflammatory skin lesions [65]. 

The exact mechanism of antibody production as well as the cascade by which gastrointestinal inflammation translates into cutaneous disease is not known. A recent study [66] revealed that the cutaneous IgA deposits present in DH skin were IgA1. Furthermore, whereas normal gut secretions contained more IgA2, gut secretions from patients with DH were predominantly IgA1. These studies suggested that IgA1 antibodies could be of gut origin, linking closely the IgA immune response in the gut to the IgA deposits in DH skin. Moreover, circulating autoantibodies against tTG and eTG appear both related to the degree of enteropathy, suggesting that the gut is the site where autoimmune response occurs. 

Definitively, the mucosal immune response to gluten could lead to IgA antibodies of mucosal origin, which persist in circulation, and a specific group of these antibodies, namely IgA anti-eTG, are able to deposit into the skin. However, the mechanism of that binding remains unknown [66]. 

It has been hypothesized that eTG is released from keratinocytes and drops to basement membrane in response to trauma; another hypothesis is that preformed circulating complex of IgA and eTG deposit in the papillary dermis [67]. Evidence of the presence of these circulating complexes is shown by the precipitation of these complexes in vessel walls of some patients with DH; however, other studies failed to demonstrate an increase of circulating immunocomplexes in DH sera [68]. 

## 5. Immune Response 

In the recent literature, many studies have attempted to clarify the exact mechanism through which gastrointestinal sensitivity results into the development of the specific lesions of DH, but such mechanism is not already completely understood [66]. 

The skin lesions of DH are characterized by the presence of an inflammatory infiltrate mainly composed by neutrophils, localized at the dermal papillary tips, the region where the cutaneous IgA deposits are found [69]. A recent histopathological study of patients with DH showed that in nearly 40% of the biopsies, only a lymphocytic infiltrate with fibrosis and ectatic capillaries were seen [70]. The initial inflammatory event is variable edema in the papillary dermis with discrete subepidermal vacuolar alteration and neutrophils along the DEJ. As the lesion develops, neutrophils, to a lesser extent eosinophils, and fibrin accumulate within the dermal papillae and form microabscesses. These become confluent, resulting in a subepidermal blister. It has been demonstrated that split formation occurs within the lamina lucida of the basement membrane zone (BMZ) [71]. 

The predominant neutrophilic nature of the disease is confirmed by the improvement of the skin lesions after the somministration of dapsone, which inhibits neutrophil function. It has been demonstrated that neutrophils from DH patients with ongoing skin lesions showed increased expression of CD11b, a slight decreased expression of L-selectin, and increased function of the FcIgA receptor, all suggesting partial priming of the neutrophils [72]. IL-8 (CXCL8) is a potent chemoattractant and activator of neutrophils; it also causes changes in surface adhesion protein expression in neutrophils by inducing the shedding of L-selectin and upregulation of CD11b/CD18 binding activity [73]. It has previously demonstrated that patients with DH have increased levels of serum IL-8 [74], but the source of this increase is not known yet. Although elevated levels of serum IL-8 are seen in patients with active skin disease, IL-8 is also elevated in patients who are on gluten-containing diets but have no active skin lesions owing to therapy with dapsone. These observations suggest that the gastrointestinal mucosa, and not inflamed epidermis, may be the source of IL-8 [75]. 

However, small bowel biopsy from patients with isolated CD or in CD associated with DH shows that neutrophils are not the predominant cells; it is possible that neutrophils represent the earliest inflammatory cell, but the continued stimulation with gluten results in a transition to the more typical mononuclear cell infiltrate. Confirming this hypothesis, it has been demonstrated a pronounced neutrophilic activation in CD after rectal gluten challenge in the first 4–6 hours [76, 77]. 

Skin lesions are predominantly associated with characteristic areas (extensor surfaces, elbows, knee, and buttocks) that are the site of constant minor trauma. 

Even if sun exposure can improve skin lesions in DH patients, it has been postulated that minor trauma and UVB irradiation to the skin may lead to the expression of critical adhesion molecules on epidermal endothelial cells (E-selectin) and proinflammatory cytokines such as IL-8, which would predispose these areas to the development of skin lesions [78, 79]. These events are required to lead the chemotaxis of the partially primed neutrophils (with an activated Fc IgA receptor to move into the skin) from the microvessels and their progressive accumulation at the top of the dermal papillae [80]. In microtraumatic areas, the neutrophils, through Fc fragment of IgA receptor, bind to this immunoglobulin and then can release lysosomial enzymes such as proteases and increase their phagocytic activity. Proteases, together with collagenases and stromelysin 1 released by keratinocytes, are essential to the development of the blister. Moreover, apoptosis, that has been shown to be increased in DH basal keratinocytes, could contribute to the development of DH skin lesions. Using TUNEL technique, we demonstrated an early activation of programmed cell death in perilesional DH skin with respect to healthy specimens, while Bax/Bcl-2 ratio was almost the same in the epidermis of perilesional/lesional DH and healthy skin specimens. In DH, both Bax and Bcl-2 proteins were increased in the dermal perivascular compartment. Fas showed a prevalently epidermal staining, while FasL was distributed in perivascular and subjunctional dermis; some FasL+ cells infiltrated the DEJ and the basal layer of epidermis [81]. 

Together with the neutrophils and, to a lesser extent, the eosinophils, other immune cell populations have been implicated in the skin damage. In particular, T cells are always detected both in perilesional and lesional skin of DH patients, suggesting their role in triggering the inflammatory response. Accordingly, T cells found in DH skin, that mainly belong to the Th2 pattern, are able to express IL-4 and IL-5, inflammatory cytokines that can activate mast cells and endothelial cells, and can enhance neutrophil and eosinophil recruitment, amplifying the skin inflammatory process [82]. We previously demonstrated a strong extracellular staining with anti-IL-4 monoclonal antibody in the upper dermis with a prevalent perivascular pattern in perilesional areas, whereas in the dermal-epidermal separation sites, there was an intense, scattered distribution. IL-5 was intensely expressed, mainly at the intracellular level, by eosinophils and lymphocytes, the staining intensity of this cytokine correlating with the number of infiltrating cells. Moreover, an intense expression of HLA-DR was detected not only on basal keratinocytes but also on dermal papillary and subpapillary infiltrate and endothelium.

To summarize, the development of skin lesions in DH could depend upon an ongoing mucosal immune response in the gut associated with elevated IL-8 levels and the production of a sufficient local concentration gradient of IL-8 and other chemokines (perhaps as a result of local trauma) that would attract “partially primed neutrophils” to the skin. The presence of IgA in the skin of patients with DH and the presence of IgA receptors on the surface of neutrophils suggest that IgA may function as an additional proinflammatory stimulus. 

## 6. Conclusions 

The pathogenic mechanism underlying DH is multifactorial, involving genetic, environmental, and autoimmune factors. A potential explanation is that in susceptible individuals (HLA association) the development of skin lesions is related to an active chronic gastrointestinal mucosal inflammation as a result of a persistent gluten challenge, with a local immune response and the production of mucosal IgA. A part of circulating IgA (anti-eTG) binds to the skin. Consequently, the gastrointestinal mucosal immune response results in increased levels of circulating cytokines which may partially prime neutrophils as well as active Th2 and endothelial cells. UVB and minor microtrauma to the skin increase local cytokines production, leading to the egress of neutrophils, deposition of IgA at the DEJ, and thus to the development of DH skin lesions. 

## References

[1] Kárpáti S (2012). Dermatitis herpetiformis. *Clinics in Dermatology*.

[2] Bolotin D, Petronic-Rosic V (2011). Dermatitis herpetiformis: part I. Epidemiology, pathogenesis, and clinical presentation. *Journal of the American Academy of Dermatology*.

[3] Kaukinen K, Collin P, Mäki M (2007). Latent coeliac disease or coeliac disease beyond villous atrophy?. *Gut*.

[4] Troncone R, Jabri B (2011). Coeliac disease and gluten sensitivity. *Journal of Internal Medicine*.

[5] Bolotin D, Petronic-Rosic V (2011). Dermatitis herpetiformis: part II. Diagnosis, management, and prognosis. *Journal of the American Academy of Dermatology*.

[6] Fry L (2002). Dermatitis herpetiformis: problems, progress and prospects. *European Journal of Dermatology*.

[7] Yeh SW, Ahmed B, Sami N, Ahmed AR (2003). Blistering disorders: diagnosis and treatment. *Dermatologic Therapy*.

[8] Nicolas MEO, Krause PK, Gibson LE, Murray JA (2003). Dermatitis herpetiformis. *International Journal of Dermatology*.

[9] Fabbri P, Caproni M Dermatitis herpetiformis. http://www.orpha.net/data/patho/GB/uk-DermatitisHerpetiformis.pdf.

[10] Caproni M, Antiga E, Melani L, Fabbri P (2009). Guidelines for the diagnosis and treatment of dermatitis herpetiformis. *Journal of the European Academy of Dermatology and Venereology*.

[11] Ko CJ, Colegio OR, Moss JE, McNiff JM (2010). Fibrillar IgA deposition in dermatitis herpetiformis—an underreported pattern with potential clinical significance. *Journal of Cutaneous Pathology*.

[12] Shibahara M, Nanko H, Shimizu M (2002). Dermatitis herpetiformis in Japan: an update. *Dermatology*.

[13] Ingen-Housz-Oro S (2011). Dermatitis herpetiformis: a review. *Annales de Dermatologie et de Venereologie*.

[14] Sárdy M, Tietze J (2009). Dermatitis herpetiformis:An update of the pathogenesis. *Hautarzt*.

[15] Spurkland A, Ingvarsson G, Falk ES, Knutsen I, Sollid LM, Thorsby E (1997). Dermatitis herpetiformis and celiac disease are both primarily associated with the HLA-DQ (*α*1∗0501, *β*1∗02) or the HLA-DQ (*α*1∗03, *β*1∗0302) heterodimers. *Tissue Antigens*.

[16] Marietta E, Black K, Camilleri M (2004). A new model for dermatitis herpetiformis that uses HLA-DQ8 transgenic NOD mice. *Journal of Clinical Investigation*.

[17] Sachs JA, Awad J, McCloskey D (1986). Different HLA associated gene combinations contribute to susceptibility for coeliac disease and dermatitis herpetiformis. *Gut*.

[18] Katz SI, Hertz KC, Rogentine GN, Strober W (1977). HLA-B8 and dermatitis herpetiformis in patients with IgA deposits in skin. *Archives of Dermatology*.

[19] Katz SI, Falchuk ZM, Dahl MV, Rogentine GN, Strober W (1972). HL-A8: a genetic link between dermatitis herpetiformis and gluten-sensitive enteropathy. *Journal of Clinical Investigation*.

[20] Karpati S, Kosnai I, Verkasalo M (1986). HLA antigens, jejunal morphology and associated diseases in children with dermatitis herpetiformis. *Acta Paediatrica Scandinavica*.

[21] Hall RP, Sanders ME, Duquesnoy RJ, Katz SI, Shaw S (1989). Alterations in HLA-DP and HLA-DQ antigen frequency in patients with dermatitis herpetiformis. *Journal of Investigative Dermatology*.

[22] Tokunaga K, Juji T (1990). Distribution of MHC alleles in Japanese. *Nippon Rinsho*.

[23] Hervonen K, Karell K, Holopainen P, Collin P, Partanen J, Reunala T (2000). Concordance of dermatitis herpetiformis and celiac disease in monozygous twins. *Journal of Investigative Dermatology*.

[24] Hervonen K, Hakanen M, Kaukinen K, Collin P, Reunala T (2002). First-degree relatives are frequently affected in coeliac disease and dermatitis herpetiformis. *Scandinavian Journal of Gastroenterology*.

[25] Van Belzen MJ, Meijer JWR, Sandkuijl LA (2003). A major non-HLA locus in celiac disease maps to chromosome 19. *Gastroenterology*.

[26] Sánchez E, Alizadeh BZ, Valdigem G (2007). MYO9B gene polymorphisms are associated with autoimmune diseases in Spanish population. *Human Immunology*.

[27] Monsuur AJ, Bakker PIWD, Alizadeh BZ (2005). Myosin IXB variant increases the risk of celiac disease and points toward a primary intestinal barrier defect. *Nature Genetics*.

[28] Koskinen LLE, Korponay-Szabo IR, Viiri K (2008). Myosin IXB gene region and gluten intolerance: linkage to coeliac disease and a putative dermatitis herpetiformis association. *Journal of Medical Genetics*.

[29] Leonard J, Haffenden G, Tucker W (1983). Gluten challenge in dermatitis herpetiformis. *New England Journal of Medicine*.

[30] Lioger B, Machet MC, Machet L (2010). Dermatitis herpetiformis. *Presse Medicale*.

[31] Fasano A (2009). Surprise from celiac disease. *Scientific American*.

[32] Matthias T, Pfeiffer S, Selmi C, Gershwin ME (2010). Diagnostic challenges in celiac disease and the role of the tissue transglutaminase-neo-epitope. *Clinical Reviews in Allergy and Immunology*.

[33] Schuppan D (2000). Current concepts of celiac disease pathogenesis. *Gastroenterology*.

[34] Shan L, Molberg O, Parrot I (2002). Structural basis for gluten intolerance in Celiac Sprue. *Science*.

[35] Skovbjerg H, Noren O, Anthonsen D, Moller J, Sjöström H (2002). Gliadin is a good substrate of several transglutaminases: possible implication in the pathogenesis of coeliac disease. *Scandinavian Journal of Gastroenterology*.

[36] Skovbjerg H, Koch C, Anthonsen D, Sjöström H (2004). Deamidation and cross-linking of gliadin peptides by transglutaminases and the relation to celiac disease. *Biochimica et Biophysica Acta*.

[37] Fleckenstein B, Molberg Ø, Qiao SW (2002). Gliadin T cell epitope selection by tissue transglutaminase in celiac disease. Role of enzyme specificity and pH influence on the transamidation versus deamidation reactions. *Journal of Biological Chemistry*.

[38] Fleckenstein B, Qiao SW, Larsen MR, Jung G, Roepstorff P, Sollid LM (2004). Molecular characterization of covalent complexes between tissue transglutaminase and gliadin peptides. *Journal of Biological Chemistry*.

[39] Ciccocioppo R, Di Sabatino A, Ara C (2003). Gliadin and tissue transglutaminase complexes in normal and coeliac duodenal mucosa. *Clinical and Experimental Immunology*.

[40] Nehra V (1998). New clinical issues in celiac disease. *Gastroenterology Clinics of North America*.

[41] Mantzaris G, Jewell DP (1991). In vivo toxicity of a synthetic dodecapeptide from A gliadin in patients with coeliac disease. *Scandinavian Journal of Gastroenterology*.

[42] De Ritis G, Auricchio S, Jones HW, Lew EJL, Bernardin JE, Kasarda DD (1988). In vitro (organ culture) studies of the toxicity of specific A-gliadin peptides in celiac disease. *Gastroenterology*.

[43] Dieterich W, Esslinger B, Trapp D (2006). Cross linking to tissue transglutaminase and collagen favours gliadin toxicity in coeliac disease. *Gut*.

[44] Turchin I, Barankin B (2005). Dermatitis herpetiformis and gluten-free diet. *Dermatology Online Journal*.

[45] Garioch JL, Lewis HM, Sargent I, Leonard JN, Fry L (1994). 25 years’ experience of a gluten-free diet in the treatment of dermatitis herpetiformis. *British Journal of Dermatology*.

[46] Fabbri P *Immunità e Cute. Le Dermatosi a Patogenesi Immunologica*.

[47] Calabuig M, Torregosa R, Polo P (1990). Serological markers and celiac disease: a new diagnostic approach?. *Journal of Pediatric Gastroenterology and Nutrition*.

[48] Dieterich W, Ehnis T, Bauer M (1997). Identification of tissue transglutaminase as the autoantigen of celiac disease. *Nature Medicine*.

[49] Caputo I, Barone MV, Martucciello S, Lepretti M, Esposito C (2009). Tissue transglutaminase in celiac disease: role of autoantibodies. *Amino Acids*.

[50] Kárpáti S (2004). Dermatitis herpetiformis: close to unravelling a disease. *Journal of Dermatological Science*.

[51] James SP (2005). Prototypic disorders of gastrointestinal mucosal immune function: celiac disease and Crohn’s disease. *Journal of Allergy and Clinical Immunology*.

[52] Kumar V, Jarzabek-Chorzelska M, Sulej J, Rajadhyaksha M, Jablonska S (2001). Tissue transglutaminase and endomysial antibodies-diagnostic markers of gluten-sensitive enteropathy in dermatitis herpetiformis. *Clinical Immunology*.

[53] Rose C, Armbruster FP, Ruppert J, Igl BW, Zillikens D, Shimanovich I (2009). Autoantibodies against epidermal transglutaminase are a sensitive diagnostic marker in patients with dermatitis herpetiformis on a normal or gluten-free diet. *Journal of the American Academy of Dermatology*.

[54] Caproni M, Cardinali C, Renzi D, Calabró A, Fabbri P (2001). Tissue transglutaminase antibody assessment in dermatitis herpetiformis [9]. *British Journal of Dermatology*.

[55] Tursi A, Brandimarte G, Giorgetti GM (2003). Prevalence of antitissue transglutaminase antibodies in different degrees of intestinal damage in celiac disease. *Journal of Clinical Gastroenterology*.

[56] Sardy M, Karpati S, Merkl B (2002). Epidermal transglutaminase (Tgase 3) is the autoanitgen of dermatitis herpetiformis. *Journal of Experimental Medicine*.

[57] Lorand L, Graham RM (2003). Transglutaminases: crosslinking enzymes with pleiotropic functions. *Nature Reviews Molecular Cell Biology*.

[58] Hitomi K (2005). Transglutaminases in skin epidermis. *European Journal of Dermatology*.

[59] Donaldson MR, Zone JJ, Schmidt LA (2007). Epidermal transgluaminase deposits in perilesional and uninvolved skin in patients with dermatitis herpetiformis. *Journal of Investigative Dermatology*.

[60] Marietta EV, Camilleri MJ, Castro LA, Krause PK, Pittelkow MR, Murray JA (2008). Transglutaminase autoantibodies in dermatitis herpetiformis and celiac sprue. *Journal of Investigative Dermatology*.

[61] Heil PM, Volc-Platzer B, Karlhofer F (2005). Transglutaminases as diagnostically relevant autoantigens in patients with gluten sensitivity. *Journal of the German Society of Dermatology*.

[62] Hull CM, Liddle M, Hansen N (2008). Elevation of IgA anti-epidermal transglutaminase antibodies in dermatitis herpetiformis. *British Journal of Dermatology*.

[63] Zone JJ, Schmidt LA, Taylor TB (2011). Dermatitis herpetiformis sera or goat anti-transglutaminase-3 transferred to human skin-grafted mice mimics dermatitis herpetiformis immunopathology. *Journal of Immunology*.

[64] Antiga E, Caproni M, Fabbri P (2011). Comment on “dermatitis herpetiformis sera or goat anti- transglutaminase-3 transferred to human skin-grafted mice mimics dermatitis herpetiformis immunopathology”. *Journal of Immunology*.

[65] Cardones ARG, Hall RP (2011). Pathophysiology of dermatitis herpetiformis: a model for cutaneous manifestations of gastrointestinal inflammation. *Dermatologic Clinics*.

[66] Sárdy M, Kárpáti S, Merkl B, Paulsson M, Smyth N (2002). Epidermal transglutaminase (TGase 3) is the autoantigen of dermatitis herpetiformis. *Journal of Experimental Medicine*.

[67] Preisz K, Sárdy M, Horváth A, Kárpáti S (2005). Immunoglobulin, complement and epidermal transglutaminase deposition in the cutaneous vessels in dermatitis herpetiformis. *Journal of the European Academy of Dermatology and Venereology*.

[68] Hall RP (1992). Dermatitis herpetiformis. *Journal of Investigative Dermatology*.

[69] Warren SJP, Cockerell CJ (2002). Characterization of a subgroup of patients with dermatitis herpetiformis with nonclassical histologic features. *American Journal of Dermatopathology*.

[70] Smith JB, Taylor TB, Zone JJ (1992). The site of blister formation in dermatitis herpetiformis is within the lamina lucida. *Journal of the American Academy of Dermatology*.

[71] Cardones ARG, Hall RP (2011). Pathophysiology of dermatitis herpetiformis: a model for cutaneous manifestations of gastrointestinal inflammation. *Dermatologic Clinics*.

[72] Smith AD, Streilein RD, Hall RP (2002). Neutrophil CD11b, L-selectin and Fc IgA receptors in patients with dermatitis herpetiformis. *British Journal of Dermatology*.

[73] Mukaida N, Harada A, Matsushima K (1998). Interleukin-8 (IL-8) and monocyte chemotactic and activating factor (MCAF/MCP-1), chemokines essentially involved in inflammatory and immune reactions. *Cytokine and Growth Factor Reviews*.

[74] Hall RP, Takeuchi F, Benbenisty KM, Streilein RD (2006). Cutaneous endothelial cell activation in normal skin of patients with dermatitis herpetiformis associated with increased serum levels of IL-8, sE-selectin, and TNF-*α*. *Journal of Investigative Dermatology*.

[75] Hall RP, Benbenisty KM, Mickle C, Takeuchi F, Streilein RD (2007). Serum IL-8 in patients with dermatitis herpetiformis is produced in response to dietary gluten. *Journal of Investigative Dermatology*.

[76] Loft DE, Marsh MN, Sandle GI (1989). Studies of intestinal lymphoid tissue. XII. Epithelial lymphocyte and mucosal responses to rectal gluten challenge in celiac sprue. *Gastroenterology*.

[77] Kristjansson G, Serra J, Lööf L, Venge P, Hällgren R (2005). Kinetics of mucosal granulocyte activation after gluten challenge in coeliac disease. *Scandinavian Journal of Gastroenterology*.

[78] Strickland I, Rhodes LE, Flanagan BF, Friedmann PS (1997). TNF-*α* and IL-8 are upregulated in the epidermis of normal human skin after UVB exposure: correlation with neutrophil accumulation and E-selectin expression. *Journal of Investigative Dermatology*.

[79] Takeuchi F, Streilein RD, Hall RP (2003). Increased E-selectin, IL-8 and IL-10 gene expression in human skin after minimal trauma. *Experimental Dermatology*.

[80] Alonso-llamazares J, Gibson LE, Rogers RS (2007). Clinical, pathologic, and immunopathologic features of dermatitis herpetiformis: review of the Mayo Clinic experience. *International Journal of Dermatology*.

[81] Caproni M, Torchia D, Antiga E (2005). The role of apoptosis in the pathogenesis of dermatitis herpetiformis. *International Journal of Immunopathology and Pharmacology*.

[82] Caproni M, Feliciani C, Fuligni A (1998). Th2-like cytokine activity in dermatitis herpetiformis. *British Journal of Dermatology*.

